# Early metabolic and hemodynamic indicators of kidney dysfunction in mice offspring from parental low protein diet

**DOI:** 10.1186/s40348-024-00184-8

**Published:** 2024-10-16

**Authors:** Fabiola Diniz, Francesca Edgington-Giordano, Samir S. El-Dahr, Giovane G. Tortelote

**Affiliations:** Section of Pediatric Nephrology, Department of Pediatrics, Tulane University School of Medicine, New Orleans, LA 70112 USA

**Keywords:** Kidney, Low protein diet, Developmental programming, Nephron endowment, Hypertension, Oligonephropathy, Metabolism, Hemodynamic changes

## Abstract

**Background:**

Parental malnutrition, particularly a low-protein diet (LPD), causes oligonephropathy at birth and predisposes offspring to hypertension and chronic kidney disease later in life. The onset of adult kidney disease varies based on genetics and environmental factors, often with subclinical alterations in kidney function being overlooked. This study aimed to examine changes in kidney morphology before significant kidney function decline in the offspring of mice fed a low-protein diet.

**Methods:**

Using a combination of histological analysis, kidney metabolic and hemodynamic panel assessments, and advanced statistical techniques such as Linear Discriminant Analysis (LDA) and Principal Component Analysis (PCA), we investigated the initial impact of a maternal low-protein diet (LPD) on kidney development and function. Our study utilized 12-week-old F1 mice from F0 parents fed either a low-protein diet (LPD) or a normal-protein diet (NPD) before the onset of hypertension.

**Results:**

The offspring (F1 generation) of parents (F0 generation) fed an LPD show reduced body weight from birth to P20. The kidney weight was also reduced compared to F1 offspring from parents fed an NPD. At 12 weeks of age, body weight normalized, but kidney weight remained low. Offspring of parents fed an LPD displayed abnormal kidney morphology, including dilated tubules, oligonephropathy, and fluid-filled cysts which had worsened with age. A kidney metabolic panel analysis at 12 weeks revealed a slight but consistent increase in urine albumin, plasma creatinine, mean urea, and BUN concentrations. Although no significant changes in hemodynamic variables were observed, 2/12 mice, both males, showed alterations in systolic blood pressure, suggesting sex-specific effects when comparing F1 mice from F0 fed either diet. Overall, kidney metabolic changes were strongly correlated to parental LPD.

**Conclusion:**

Our findings indicate that significant kidney damage must accumulate in the F1 generation from parents fed an LPD before any detectable changes in blood pressure occur. Our study suggests that small variations in kidney metabolic function may point to early kidney damage and should not be overlooked in the offspring of these malnourished mice and likely humans.

**Supplementary Information:**

The online version contains supplementary material available at 10.1186/s40348-024-00184-8.

## Introduction

The kidney plays a crucial role in the body by eliminating toxic wastes like urea and creatinine, and reabsorbing essential molecules such as proteins, sugars, and micronutrients. Additionally, the kidneys regulate blood volume, blood pressure, osmolarity, and pH balance. They are also responsible for producing erythropoietin and vitamin D. Chronic kidney disease is a slow and cumulative process that disrupts these vital functions and affects about 15% of the world’s population, while hypertension impacts twice as many people [1]. Thus, any detectable change in blood pressure is usually a sign that kidneys are no longer capable of performing their functions efficiently [2–4].

Intrauterine Growth Restriction (IUGR) is a condition where a fetus in the womb does not grow at the expected rate during pregnancy. This can result in the fetus being smaller than expected for its gestational age. Various factors, including placental problems, genetic conditions, or infections can cause IUGR. Maternal health issues like high blood pressure, or malnutrition can also cause IUGR [5–11]. Previous studies have shown that offspring from IUGR pregnancies, particularly those born to parents fed an LPD, frequently exhibit metabolic dysfunctions, including disrupted lipid and glucose homeostasis and hypertension [12–18]. These findings underscore the potential cumulative impact of poor prenatal nutrition on renal and cardiovascular health.

IUGR hampers normal kidney development and increases the risk for hypertension, and kidney disease later in life [19–23]. Additionally, it has been demonstrated that the prenatal stimuli, particularly the maternal diet, plays a crucial role in shaping the health development of the offspring, influencing susceptibility to renal and cardiovascular problems later in life [19, 24].

Rodent models have been used to demonstrate that LPD can lead to structural and functional changes in the kidneys, including decreased nephron number, glomerulosclerosis, and interstitial fibrosis [16, 25]. We and others have shown that, in mice, an LPD during pregnancy is associated with abnormal embryonic development, reduced body, and kidney weight at birth, and oligonephropathy, which are risk factors for hypertension and chronic kidney disease [26–28].

Although kidney development defects are present, changes in blood pressure in the offspring of mice fed a low-protein diet are not detectable until 18 weeks of age, with more consistent alterations observed after 20 weeks [29, 30]. High blood pressure causes kidney damage, making it challenging to determine the exact contribution of hypertension versus diet to kidney damage observed in adult offspring from parents fed an LPD. To address this, we examined the effects of parental LPD on kidney morphology and function in 12-week-old mice, before the onset of hypertension.

## Methods

### Ethical considerations

All animal procedures were approved by the Institutional Animal Care and Use Committee (IACUC), protocol # 1558, and conducted in accordance with the National Institutes of Health guidelines for the care and use of laboratory animals.

### Diet

The low-protein diet used for this study was purchased from Envigo (TD 90016). The low-protein diet is isocaloric and contains 6.1% protein, 75.6% carbohydrate, 5.5% fat and 3.8 Kcal/g. The control diet is Envigo TD 91,352 has 20.3% protein, 61.6% carbohydrate, 5.5% fat and 3.8 Kcal/g. The only difference between the two diets is the amount of protein, and the 6% protein diet has more sucrose and cellulose to maintain an iso-caloric formula.

### Mouse model and breeding

**Fig. 1:**
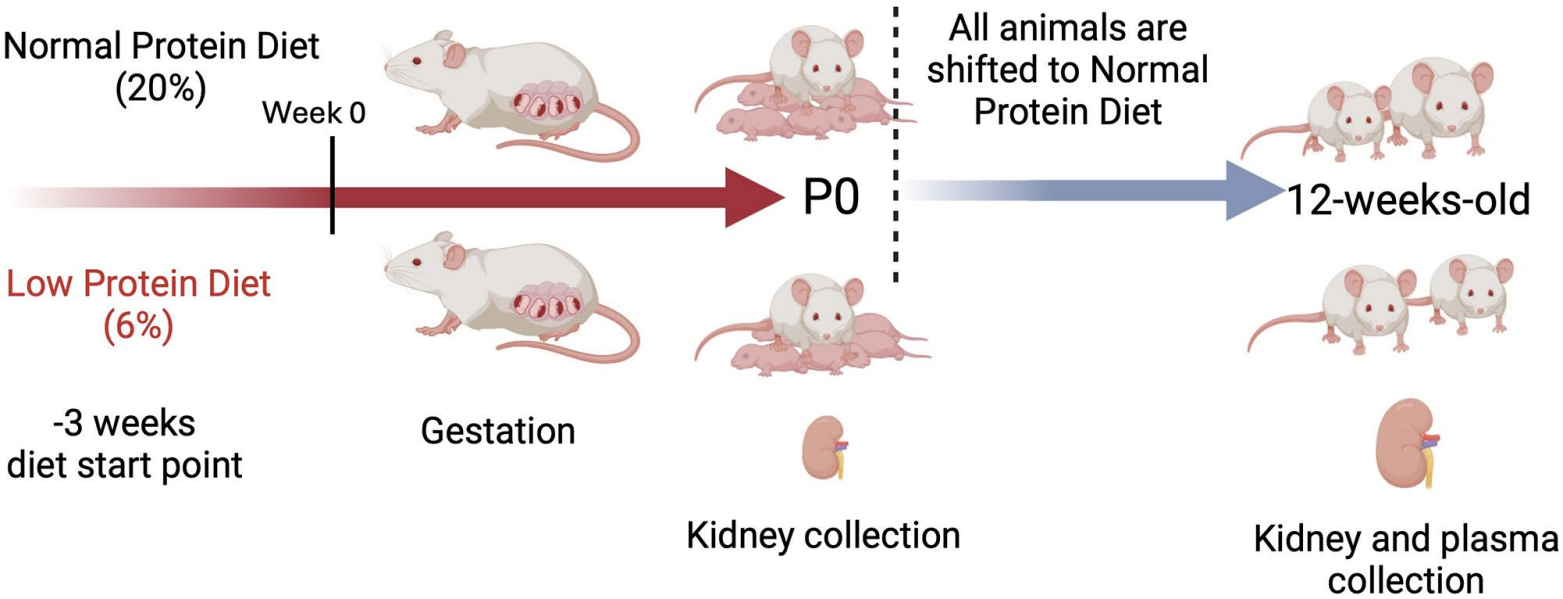
Parental LPD leads to morphological defects during kidney development. The study design shows the separation of the two diet groups (NPD and LPD). The starting point at -3 weeks indicates when the diet started to be given to the mice. The mattings were set at week 0. Pregnancy was confirmed by observation of a vaginal plug 12 h after mating. Sample collection and end points are depicted

CD1 female and male mice were purchased from the Charles River laboratory (strain code 022) and bred in-house. Male and female mice were assigned to either an NPD or a LPD for three weeks from weaning (P20) onwards, before mating. The diets were maintained throughout the pairing and pregnancy periods. Timed pregnancies are defined as female mice being paired with male mice overnight where the next day is counted as E0.5. Postnatal day zero (P0) is the day mice are born. Time points after P0 accounted for litter size and breastmilk quality. Female mice have 10 nipples for feeding so all growing litters were maintained at 10 pups or smaller, in cases of larger litters 10 pups were randomly selected at P0. This controls for postnatal food access. To control for breast milk quality, pups from 6% maternal LPD grown past P0 were fostered with a CD1 female mouse that had given birth the same day from e normal vivarium chow of 20% protein. Therefore, both LPD and control would have equal access to milk and similar milk quality postnatally. At P20 the pups were separated by diet and sex and put on a standard diet until 12 weeks. At twelve weeks of age, the animals were used for the experiments described below and Fig. 1.

### Blood pressure and pulse measurement

Tail-cuff plethysmography was utilized for the measurement of blood pressure in conscious mice from both groups, namely NPD and LPD. This procedure is deemed non-invasive, with physical restraint confined to a duration of fewer than 30 min. The apparatus was positioned within the vivarium setting to mitigate stress on the animals. Monitoring of systolic blood pressure was conducted through an automated tail-cuff volume-pressure recording system, specifically the Visitech BP-2000 Blood Pressure Analysis System. The animals underwent a two-day acclimatization period to the holder restraints, and measurements were collected over 3 consecutive days to mitigate the effects of stress induced by restraint. Each animal underwent ten to fifteen successive measurements while being gently restrained and kept at a temperature of 35 degrees Celsius.

### Measurements of metabolic features

The mice of both groups were euthanized at 12 weeks of age, according to the Tulane University IACUC guidelines. Next, blood was collected via heart puncture. At least 500 µL of blood was placed in plasma collection tubes, which were inverted 10 times to prevent coagulation. The plasma was then centrifuged, stored at -80˚C, and later used to measure creatinine (a marker of kidney filtration) and Blood Urea Nitrogen (BUN), another filtration marker. Urine was collected from the bladder, stored at -80˚C, and later used for urine albumin measurements. The blood and urine samples were sent to the University of Alabama at Birmingham (UAB-UCSD O’Brien Center for Acute Kidney Injury) for analysis.

### Morphological analysis of kidney samples

Hematoxylin and Eosin-stained kidney sections were used to visualize morphological structures of the developing and adult kidneys. A large image stitching was created to show a complete image of the kidney section and NIS elements at high resolution. Next, fully formed glomeruli were counted in sections that were 5 μm thick and 15 μm apart, (given that on average a mature glomerulus measures roughly 60–85 μm). We counted 3 mid-coronal sections that are 15 μm and averaged those counts to produce an average count per section for that kidney. The glomerular Eqidiameter value was measured as the longest diameter through the glomeruli measured. The ImageJ was used to calculate the area of each glomerulus. First, we set the scale of the image based on the magnification (µm/pixel). Then a perimeter of the glomerulus was traced using the “Freehand Selection” tool. Next, we use the “Measure” function to calculate the area inside the traced perimeter. This procedure was repeated for 10 randomly selected glomeruli per section then the average was calculated.

The morphology of the nephrogenic zone, anatomy of tubular structures, and presence of fluid-filled cysts were used to compare between diet groups, at P0 and 12 weeks of age.

### Sex-specific comparisons

Hemodynamic data were also analyzed separately for male and female mice to identify any sex-specific differences.

### Statistical analysis

Comparisons between NPD and LPD groups were conducted using the Wilcoxon test, with significance set at *p* < 0.05. The calculations were made in R programming language (4.3.3). The following libraries were used for statistical calculations and plotting: ggpubr [31], tydiverse [32].

### Correlation analysis

Correlation matrices were constructed to assess relationships between metabolic and hemodynamic parameters within each dietary group. Pearson correlation coefficients were calculated to quantify these relationships. All calculations were performed in R programing language with the library “corrplot” v0.92.

### Multivariate analysis

Linear Discriminant Analysis (LDA): LDA was performed to identify linear combinations of metabolic and hemodynamic variables that best separated the NPD and LPD groups. LD1, the first linear discriminant, was analyzed to understand group separation. LDA was performed with the library MASS v7.0. Principal Component Analysis (PCA): PCA was conducted to reduce the dimensionality of the dataset and to identify principal components that explain the maximum variance. PCA biplots were generated to visualize the separation between groups and the contributions of individual variables to the principal components. PCA was performed with the library “factoextra” v1.0.7 Data Visualization and Interpretation: The results of LDA and PCA were visualized using biplots, with ellipses representing 95% confidence intervals for each group. Arrows indicated the direction and magnitude of variable contributions.

## Results

We and others have shown that parental LPD causes oligonephropathy in mice [26, 27] and rats [15, 33–35]. Previous research has shown deteriorating kidney function with loss of blood pressure regulation occurs roughly after 18–22 weeks of age in the offspring of mice fed an LPD [29, 30].

To determine if the initial loss of kidney function in the offspring of mice fed an LPD could be detected before the onset of hypertension, we studied the morphological, metabolic and hemodynamic markers of kidney function in 12-week-old offspring from mice fed either a normal protein diet (NPD) or a low-protein diet (LPD).

To control for the effects of LPD on postnatal kidney development, at birth, the LPD litters were fostered to an NPD-fed CD1 female who had given birth on the same day. To control for food availability, all litters were kept 10 pups, randomly selected (additional pups were removed at birth). The female mouse has ten functional breast glands. The litters were weaned at P20 and fed a normal chow (20% protein) until 12 weeks. The experimental design is depicted in Fig. 1.

### Effect of parental low-protein Diet on body and kidney weight

**Fig. 2:**
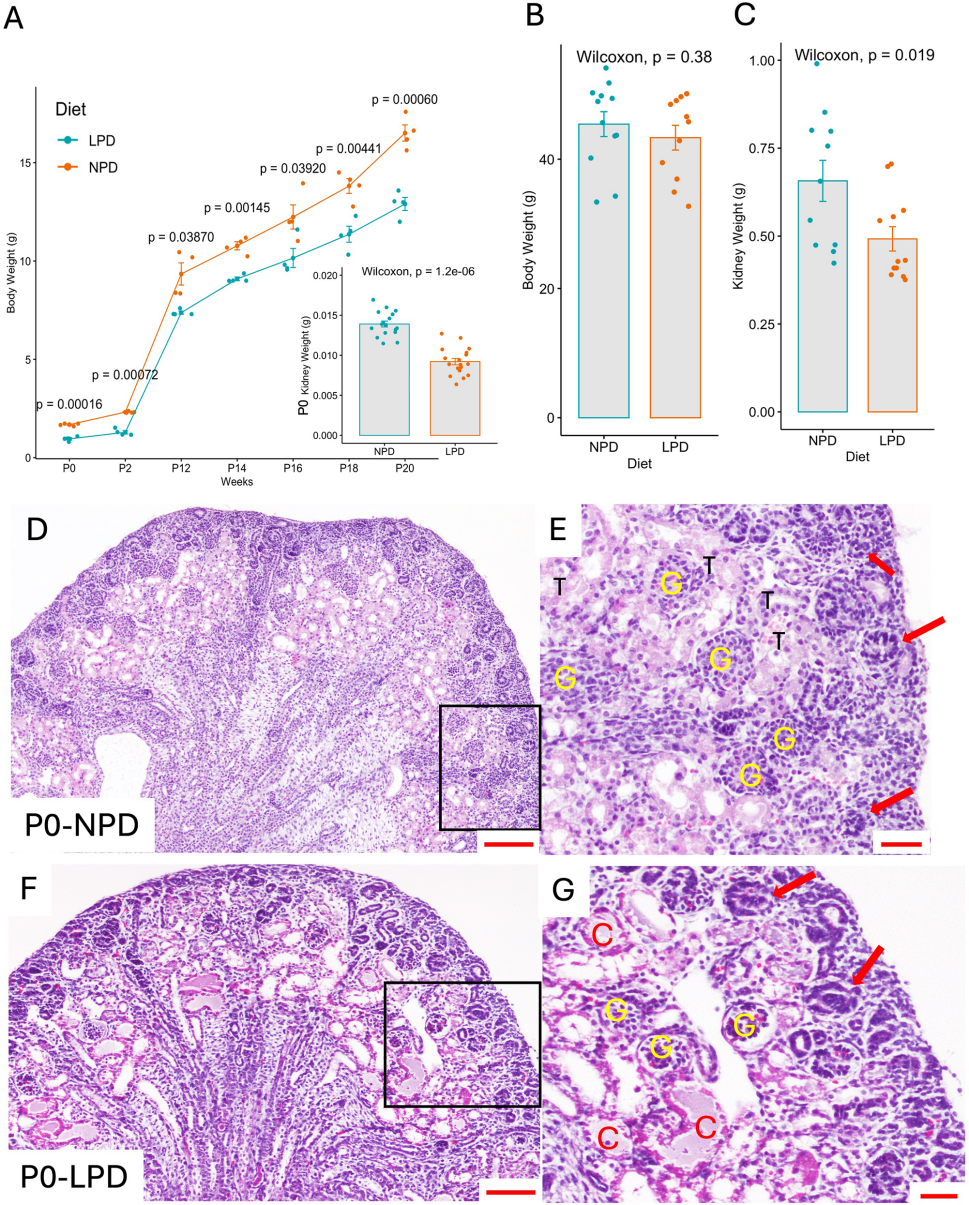
Effect of Parental Low-Protein Diet on Body and Kidney Weight, and Kidney Histology in Newborn Pups (P0) Body weight (g) growth trajectories of pups from parents fed a Normal Protein Diet (NPD, orange) and Low Protein Diet (LPD, teal) over 20 days. The graph shows a significant decrease in body weight in pups from LPD parents at multiple time points starting from P0 (*p* = 0.00016), with significant differences at later stages including P12 (*p* = 0.00145), P16 (*p* = 0.03920), and P20 (*p* = 0.00060). Inset shows kidney weight at P0, demonstrating a significant reduction in kidney weight in LPD pups (Wilcoxon, *p* = 1.2e-06) (**A**). Bar graph showing body weight (g) at 12 weeks of age for mice born to NPD and LPD parents. No significant difference in body weight between NPD and LPD mice at this age (Wilcoxon, *p* = 0.38) (**B**). Bar graph of kidney weight (g) at 12 weeks of age in NPD and LPD mice. A significant decrease in kidney weight is observed in LPD pups compared to NPD (Wilcoxon, *p* = 0.019) (**C**). Representative kidney H&E histology of P0 pups from parents fed an NPD (P0-NPD). The kidney shows a normal nephrogenic zone with several nascent nephrons and adjacent tubular structures, glomeruli can also be observed, scale bar: 100 μm (**D**). Higher magnification of the boxed region in panel D highlights normal kidney morphology with well-defined glomeruli (yellow G) and tubules (black T). Red arrows point to the nascent nephrons (renal vesicle and pretubular aggregates). Scale bar: 50 μm (**E**). Representative H&E kidney histology of P0 pup from parents fed an LPD (P0-LPD). The kidney section shows altered morphology, including abnormal nephrogenic zone, fluid-filled cysts and abnormal kidney structure such as dilated tubules, scale bar: 100 μm (**F**). Higher magnification of the boxed region in panel F reveals multiple fluid-filled cysts (red C) and altered glomeruli (yellow G) in pups from LPD-fed parents, scale bar: 50 μm (**G**)

The body weight of pups from parents fed an LPD was significantly reduced compared to pups from NPD-fed parents at multiple time points during postnatal development. The average body weight at birth (P0) of pups from parents on NPD was 1.67±0.64 g while pups from LPD parental diet weighed at an average of 0.96±0.12 g a statistically significant decrease of 42% (*p* = 0.00016). Pups from the LPD parental diet have significantly smaller kidneys than the control at birth. The average combined kidney weight of the NPD group was 0.0139±0.0017 g (*n* = 17) versus 0.0092±0.0015 g (*n* = 18) a statistically significant decrease of 35% (*p* = 1.2e-6). (Fig. 2A, inset). Notably, significant differences in body weight were observed throughout the lactation period, from P0 to P20 (Fig. 2A). Body weight growth trajectories of pups from parents fed an NPD, (orange) or LPD (teal) over 20 weeks are shown in Fig. 2A. We observed a significant decrease in body weight in pups from LPD parents at multiple time points starting from P0 (*p* = 0.00016), P2 (*p* = 0.0007) with significant differences at later stages including P12 (*p* = 0.00145), P14 (*p* = 0.00145), P16 (*p* = 0.03920), P18 (*p* = 0.00441), and P20 (*p* = 0.00060). These data were collected from 28 pups from 6 control litters and 28 pups from 5 LPD litters. At 12 weeks of age, the body weight was not significantly different between diets (NPD 45.43±6.66, *n* = 12, LPD = 43.33±6.35, *n* = 12, *p* = 0.38). However, kidney weight remained significantly lower (NPD 0.49±0.11, *n* = 10, LPD = 0.66±0.19, *n* = 12, *p* = 0.019) (Fig. 2C). The LPD affected both body weight and kidney weight in males and females in a similar way at the investigated time points (data not shown).

### Effect of parental low-protein Diet on kidney morphology at Birth

Histological analysis of kidney sections revealed that pups from the LPD group had marked morphological abnormalities. At P0, kidneys from the offspring of NPD-fed parents (P0-NPD) displayed normal renal architecture, the outer cortex is well defined with numerous nascent nephrons (Fig. 2E, red arrow), and normal glomeruli can be observed at low and high magnification (yellow G), abundant cortical tubular structure (T) can be observed adjacent the nephrogenic zone, likely proximal tubules (Fig. 2D and E). In contrast, pups from the LPD group (P0-LPD) exhibited pronounced alterations in kidney structure. P0-LPD pups exhibited an abnormal nephrogenic zone, and glomeruli (Fig. 2G, yellow G), dilated tubular structures, and fluid-filled cysts (red C) (Fig. 2F and G). These findings indicate that a parental LPD leads to significant impairments in kidney development.

### Morphological Changes in Kidney Structure and Glomerular Metrics in Offspring of Parental Low Protein Diet were Evident at 12 Weeks of Age

**Fig. 3:**
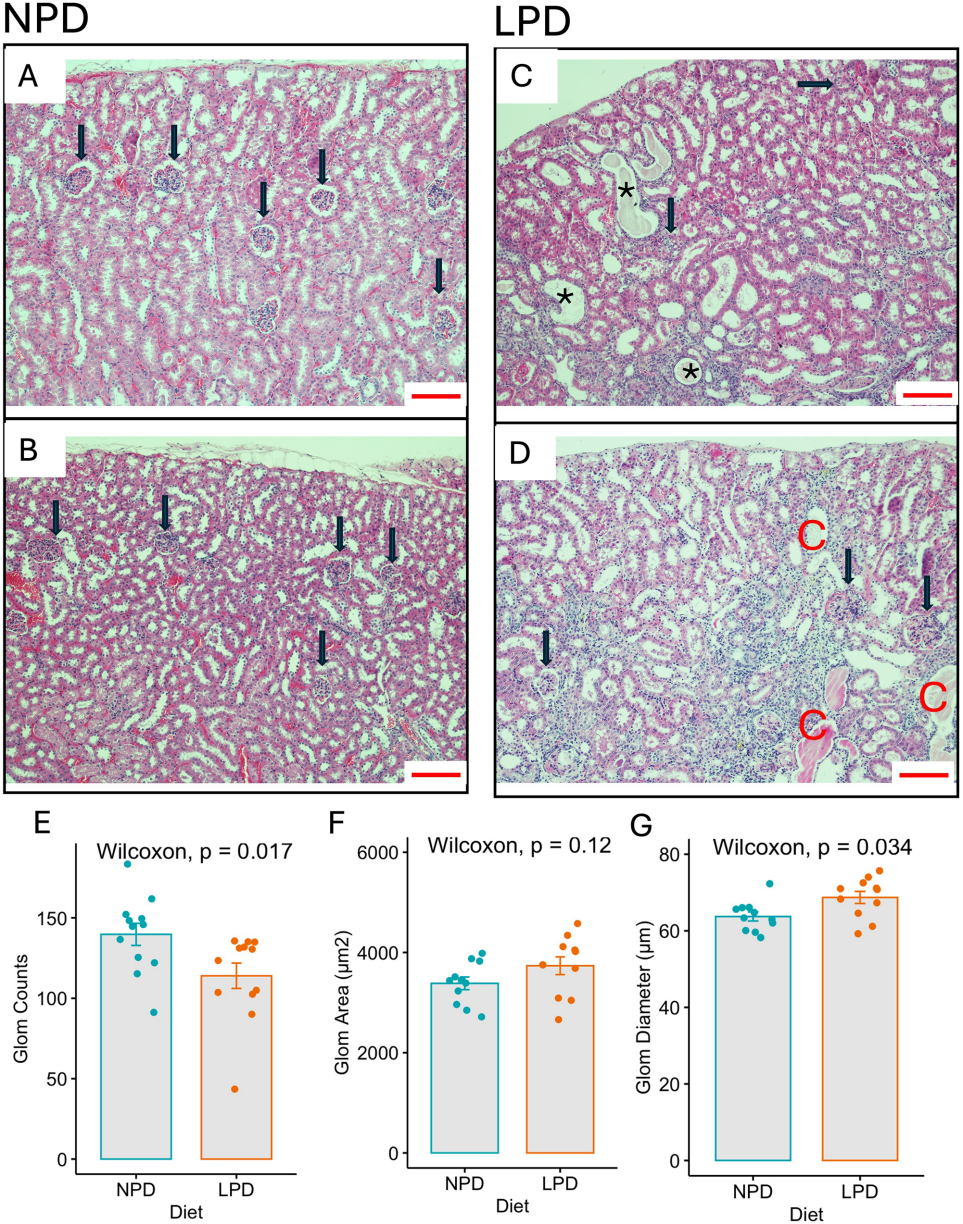
Impact of Parental Low Protein Diet on Kidney Morphology and Glomerular Metrics in 12-Week-Old Offspring. H&E staining of kidney sections from 12-week-old offspring of parents fed an NPD (**A** and **B**) or LPD (**C** and **D**). Normal glomeruli are indicated in A and B (black arrow), while LPD kidneys (C and D) have abnormal glomeruli morphology (black arrow) with dilated tubules, fluid-filled cysts (red C), abnormal glomeruli (black arrow), and abundant subcortical cells with small nuclei without expansion of extracellular matrix, suggesting immune infiltration. Quantification of glomerular counts shows a significant decrease in LPD offspring compared to NPD (Wilcoxon, *p* = 0.017 *n* = 12 mice per diet) (**E**). Comparison of the glomerular area between NPD (*n* = 11) and LPD (*n* = 10) offspring shows no significant difference (Wilcoxon, *p* = 0.12) (**F**). Glomerular diameter is significantly increased in LPD offspring, suggesting compensatory hypertrophy in response to reduced glomerular count (Wilcoxon, *p* = 0.034, *n* = 12 mice per diet) (**G**). Scale bar: 100 μm

The parental LPD resulted in significant morphological alterations in the kidneys of 12-week-old offspring compared to those from parents fed an NPD. Histological analysis of H&E stained kidney sections revealed that kidneys from NPD-fed offspring displayed normal renal architecture with well-formed glomeruli (Fig. 3A, B; black arrow). In contrast, offspring from LPD-fed parents exhibited substantial kidney abnormalities, including dilated tubules, fluid-filled cysts (Fig. 3C, D; black asterisk), and abnormal glomeruli (black arrow). Interestingly, the presence of dilated tubules may suggest a compensatory hypertrophy of the remaining nephrons.

The darker, more densely stained subcortical areas with cells scattered throughout may indicate the presence of inflammatory cells, likely lymphocytes, which could suggest an immune response or inflammation. Of note, the severity of the phenotype was variable, with some animals being more affected than others within the same litter (Supp. Figure 1). The observed variability in phenotype is expected in an outbred population, like the CD1 mice utilized in this study. Other factors, such as the number of implantation sites and placental development, may also have contributed to the more severe phenotype in some mice.

The quantification of the glomeruli further confirmed the detrimental effects of the LPD. Glomerular counts were significantly reduced in LPD offspring compared to NPD controls (NPD = 114±23.9, LPD = 145±27.3, *p* = 0.017, *n* = 12 mice per diet) (Fig. 3E), indicating impaired nephron formation. Interestingly, the glomerular area was increased but did not reach statistical significance between the two groups (NPD = 3,385±419, LPD = 3,736±588, *p* = 0.12, *n* = 11 NPD, *n* = 10 LPD) (Fig. 3F). However, the glomerular diameter was significantly increased in LPD offspring (NPD = 63.5±3.91, LPD = 68.7±5.23, *p* = 0.034, *n* = 12 mice per diet) (Fig. 3G), suggesting compensatory hypertrophy in response to the reduced glomerular count, as the remaining glomeruli may have enlarged to maintain kidney function.

These results highlight the long-term consequences of a parental low-protein diet, with offspring demonstrating significant reductions in glomerular count, increased glomerular diameter, and various structural abnormalities in kidney morphology. These findings suggest that the LPD affects renal development, leading to compensatory mechanisms that may have lasting impacts on kidney function in later life.

### Metabolic markers in NPD and LPD groups

Chronic kidney damage is a slow but cumulative process. The clearance capacity of the kidneys is usually affected before any detectable change in blood pressure [2–4]. Thus, we first analyzed kidney metabolic function before the onset of hypertension in mice born from parents fed a low-protein diet, which has been recorded after ~16 weeks of age [29, 30, 36].

**Fig. 4:**
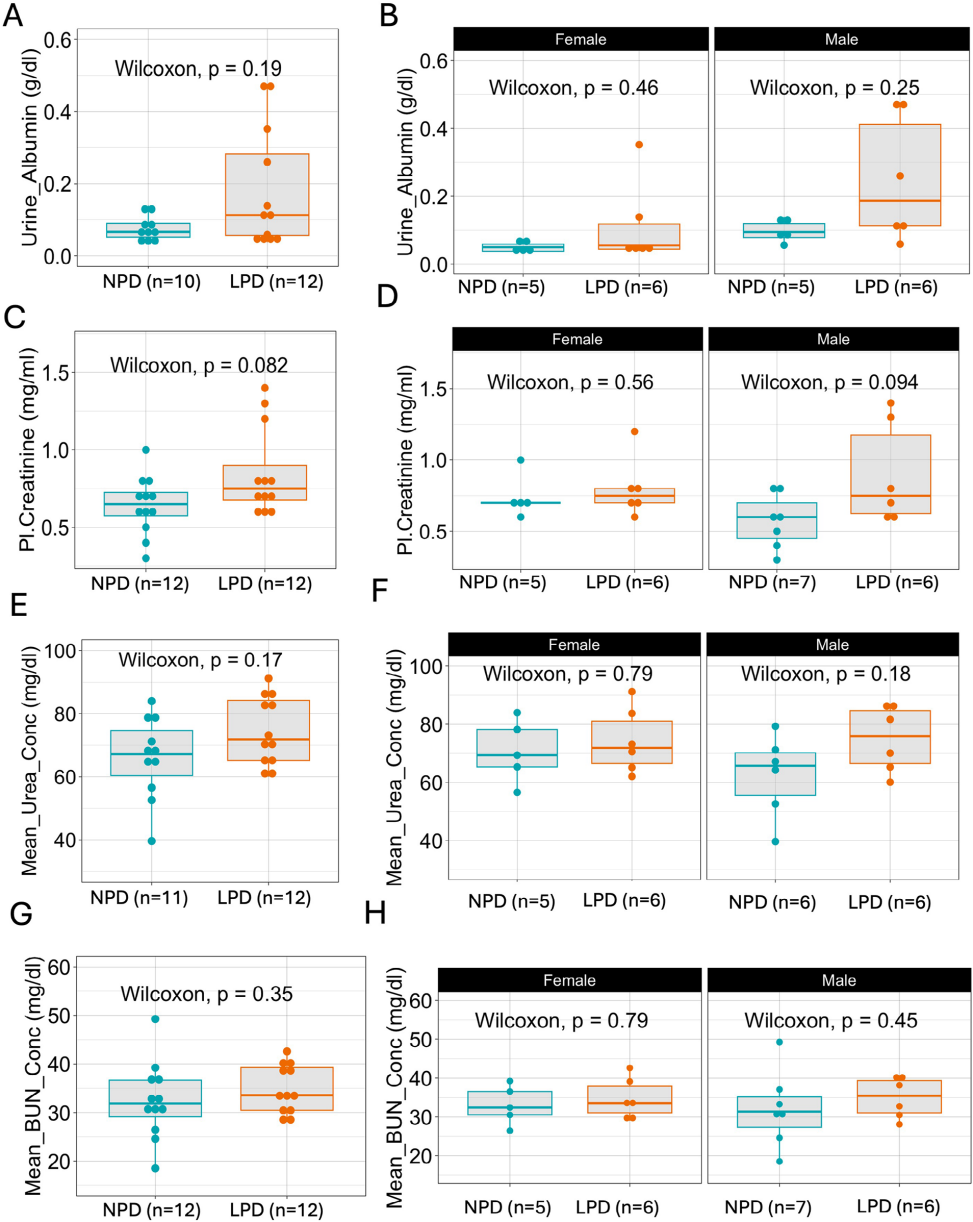
Analysis of metabolic markers in the NPD and LPD groups of 12-week-old mice. At 12 weeks urine and plasma were collected from mice of parental NPD and LPD groups. Analysis of the kidney function metabolic markers shows a trend in elevated levels of metabolites. Urine albumin (NPD 0.07 ± 0.03; LPD = 0.18 ± 0.16, p-value 0.19) (**A**), sex-specific comparisons (**B**). Plasma creatine (NPD 0.64 ± 0.18; LPD = 0.85 ± 0.28, p-value 0.08) (**C**), sex-specific comparisons (**D**); Mean Urea concentration (NPD = 69.41 ± 16.72; LPD 74.60 ± 10.72, p-value 0.17); and mean BUN concentration (NPD = 34.84 ± 4.98; LPD 32.43 ± 7.80, p-value 0.17). Statistical comparisons were carried out using the Wilcoxon test

The effects of parental dietary protein intake on kidney function were evaluated by analyzing metabolic markers in urine and plasma collected from F1 generation at 12 weeks from parents fed an NPD or LPD. The kidney metabolic panel analysis showed slight yet detectable trends of increased metabolite levels in the LPD group. Urine albumin levels were higher in the LPD group (0.18 ± 0.16) compared to the NPD group (0.07 ± 0.03), with a p-value of 0.19 (Fig. 4A). When sex-specific differences were analyzed, we observed that F1 males from parental LPD had in general higher values of urine albumin than the ones from the parental NPD group. Notice that the median of the LPD group is higher than the third quartile of the NPD group, also the interquartile range was wider in the LPD group (Fig. 4B).

Plasma creatinine levels were also elevated in the LPD offspring group (0.85 ± 0.28) relative to the NPD group (0.64 ± 0.18), with a p-value of 0.08 (Fig. 4C). Again, the same phenomenon was observed when plasma creatinine was analyzed in a sex-specific manner. F1 males from parental LPD had in general higher values of plasma creatinine than the ones from the parental NPD group. We also observed higher median values and a wider interquartile range (Fig. 4D). Additionally, mean urea (Fig. 4E and F) and BUN concentrations (Fig. 4G and H) showed slight yet detectable increases in the LPD group.

These findings indicate that at 12 weeks of age, there are trends toward increased metabolic markers of kidney function in the offspring of parents fed an LPD, suggesting potential subclinical changes in the kidneys of the offspring of the LPD group preceded the hemodynamic changes previously reported in older animals [15, 34, 37].

### Hemodynamic parameters in NPD and LPD groups

After analyzing the metabolic marker of kidney function, we decided to study whether a subclinical increase in hemodynamic parameters could also be observed in the offspring of LPD-fed mice.

**Fig. 5:**
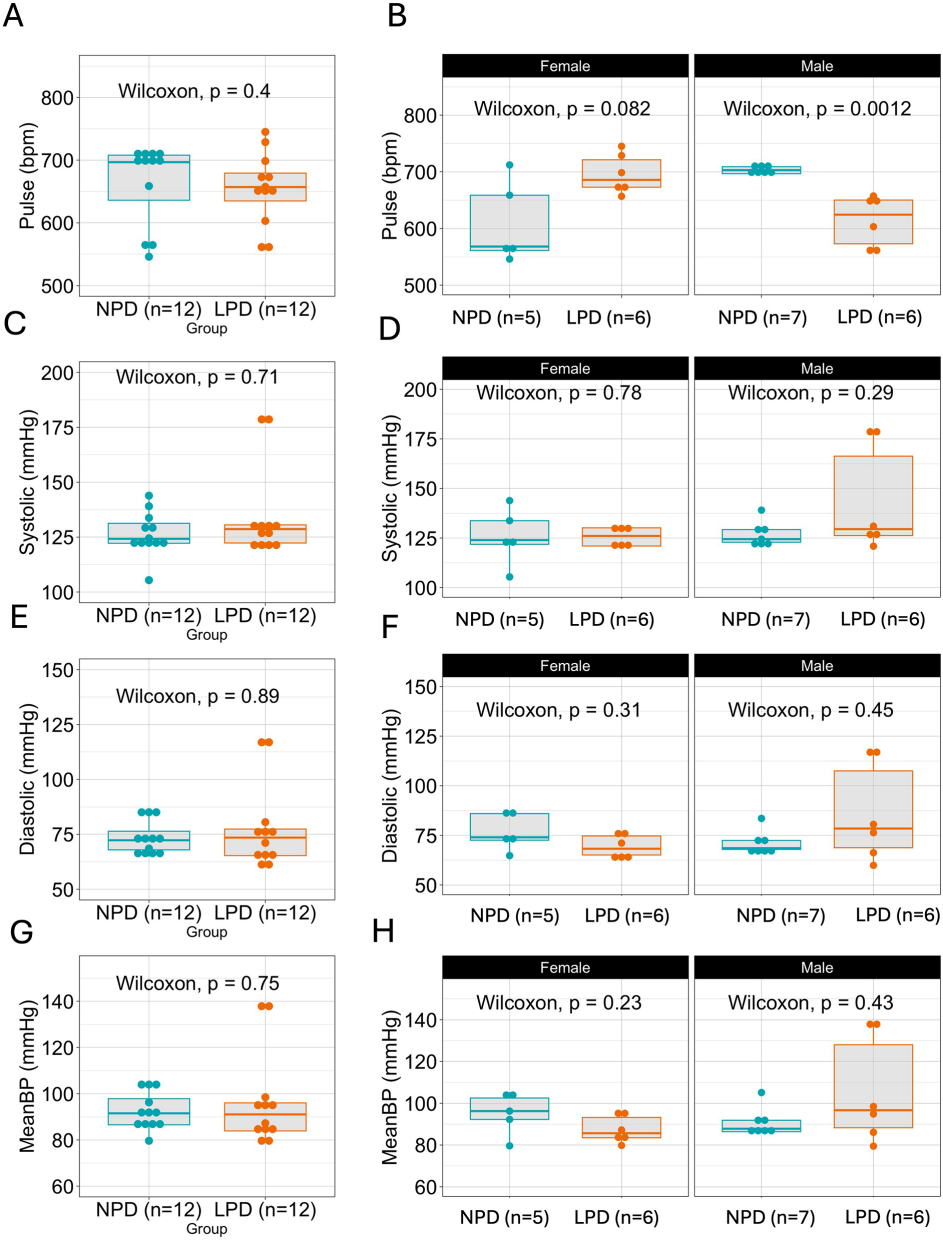
Hemodynamic panel analysis in NPD and LPD groups of 12-week-old mice. At 12 weeks mice of parental NPD and LPD groups were studied for the following hemodynamic parameters: Pulse (NPD 664 ± 65; LPD = 654 ± 57, p-value 0.4) (**A**), sex-specific comparisons (**B**). Systolic blood pressure (NPD 126.4 ± 10; LPD = 134.6 ± 20, p-value 0.71) (**C**), sex-specific comparisons (**D**); Diastolic blood pressure (NPD = 73.5 ± 7.7; LPD 77.8 ± 19.4, p-value 0.89); and mean blood pressure (NPD = 92.6 ± 8.24; LPD 96.6 ± 20.3, p-value 0.75). Statistical comparisons were carried out using the Wilcoxon test

At 12 weeks of age, the analysis of hemodynamic parameters revealed no significant differences between the offspring of mice fed NPD or LPD. Pulse rates were similar between the groups (NPD: 664 ± 65; LPD: 654 ± 57, p-value 0.4) (Fig. 5A). However, when males and females were analyzed independently. Females and males on LPD showed opposite trends (Fig. 5B). Interestingly, when only males were analyzed, there was a significantly lower pulse in the F1 offspring from the LPD-fed diet group (Fig. 5B).

Systolic blood pressure showed a slight increase in the LPD group (134.6 ± 20) compared to the NPD group (126.4 ± 10), but this was not statistically significant (p-value 0.71). Notably, 2/12 animals had values of systolic blood pressure higher than the third quartile of the group (Fig. 5C). These animals were both males. Although small, the increase in the systolic blood pressure positively correlated with the kidney metabolic panel results of the F1 offspring from the parental LPD. The Diastolic (p-value 0.89) (Fig. 5E) and mean blood pressure (p-value 0.75) (Fig. 5G) also showed no significant differences between the groups. Notably, there was a wider interquartile range when males alone were analyzed (Fig. 3F and H).

### Relationship between metabolic and hemodynamic parameters

**Fig. 6:**
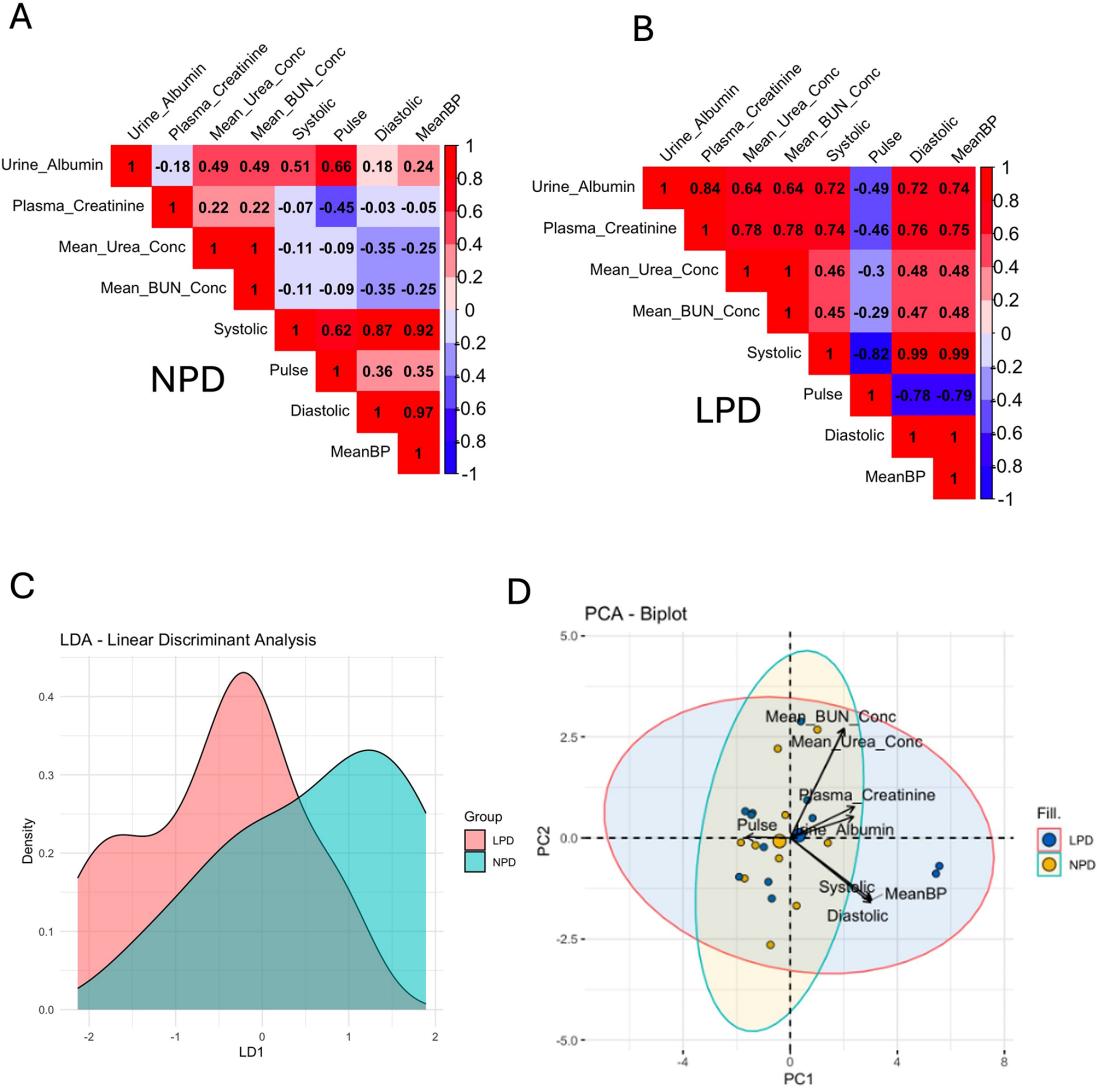
Correlation matrices comparing metabolic and hemodynamic parameters between two diets NPD and LPD. Correlation matrix between metabolic and hemodynamic variables from 12-week-old offspring of parental NPD (**A**) or parental LPD (**B**). Linear Discriminant Analysis was used to find a linear combination of features that best separates two groups (LPD and NPD). LD1 is the first linear discriminant, representing the direction that maximizes the separation between the two groups (**C**). Principal component analysis (PCA) showing PC1 and PC2 which are the first two principal components. They represent the directions of maximum variance in the data (**D**). Each point represents an individual observation (animal) in the dataset. The colors indicate the diet groups (NPD vs. LPD). The ellipses represent the 95% confidence intervals for each group (NPD and LPD). The arrows represent the original variables projected onto the PC1-PC2 plane. The direction and length of the arrows indicate the contribution of each variable to the principal components

To further understand the interaction between metabolic and cardiovascular health, we performed a correlation analysis of metabolic and hemodynamic variables in mice from both dietary groups (Fig. 6A and B). Initially with the NPD group, a weak negative correlation was observed between urine albumin and plasma creatinine (-0.18). Plasma creatinine was weakly correlated with mean urea concentration (0.22) and mean BUN concentration (0.22). Mean BUN concentration and mean urea concentration had high positive correlations (1.00) as expected. Plasma creatinine, mean urea concentration, and mean BUN concentration exhibited weak negative correlations with hemodynamic parameters (Fig. 6A).

On the other hand, in the offspring of parents fed an LPD, urine albumin displayed strong positive correlations with plasma creatinine (0.84), mean urea concentration (0.64), and mean BUN concentration (0.64), as well as with systolic and diastolic blood pressure (0.72), and mean blood pressure (0.74) (Fig. 6B). Overall, there was a gain of positive correlation between metabolic and hemodynamic parameters in the F1 offspring from F0 parents fed an LPD. Mean urea and mean BUN concentration also had higher correlations with hemodynamic parameters (Fig. 6B). The correlation analysis underscores a stronger linkage between metabolic dysfunction and blood pressure regulation in the LPD group, suggesting that a parental low-protein diet has caused accelerated renal function decrease.

### Linear Discriminant Analysis (LDA) and Principal Component Analysis (PCA) indicate the changes in metabolic markers precede hemodynamic alterations

To further elucidate the differences between the NPD and LPD groups, we performed Linear Discriminant Analysis (LDA) and Principal Component Analysis (PCA) with the offspring of both diet groups at 12 weeks of age. The LDA results indicated a clear separation between the NPD and LPD groups along the first linear discriminant axis (LD1), with the LPD group showing a distinct distribution (Fig. 6D). The blue (NPD) and red (LPD) areas represent the distribution of the diet groups along the LD1. The LPD group is primarily distributed on the left side of the LD1 axis, with a peak density around − 1. The NPD Group is mainly distributed on the right side of the LD1 axis, with a peak density of around 1. There is a region of overlap between the LPD and NPD groups around the center of the LD1 axis (approximately between − 0.5 and 0.5). This indicates some shared characteristics or measurements that are not completely distinct between the two groups, as expected at this age.

The distinct peaks for each group suggest that LDA has effectively found a direction (LD1) that separates the LPD and NPD groups based on the variable used in the analysis. We used the Kolmogorov-Smirnov test to compare the density profiles of the two diets. The Kolmogorov-Smirnov test confirmed significant differences between diets (D = 0.55, p-value 0.04). The plot indicates that LDA has successfully found a linear discriminant that can largely separate the two groups based on the underlying data. The LPD and NPD groups have different distributions along this discriminant, indicating differences in the features used for the analysis. The degree of separation suggests that the features used in the analysis can distinguish between mice on an LPD and those on an NPD. The LPD group shows stronger positive correlations among the metabolic markers (Urine Albumin, Plasma Creatinine, Mean Urea Concentration, and Mean BUN Concentration) compared to the NPD group.

Hemodynamic and metabolic parameters were positively correlated in the LPD group. This suggests that the LPD has a more pronounced effect on linking metabolic dysfunction with blood pressure regulation, indicating a possible compounding effect of the low protein diet on both renal and cardiovascular health.

We run a PCA analysis to determine the most important variables responsible for the differences between diets (Fig. 6D). NPD (yellow points) and LPD (blue points) are somewhat overlapping, indicating some similarity in the profiles of the two groups in the context of the selected variables. However, there is also some separation, suggesting that there are differences between the groups, especially along PC1. Mean BUN Concentration and Mean Urea Concentration have long arrows pointing toward the top-right quadrant. Urine albumin and Plasma Creatinine have thinner arrows pointing toward the top-right quadrant. This indicates that these variables contribute strongly to PC1 and PC2.

Systolic, Diastolic, and Mean blood pressure have arrows pointing toward the bottom-right quadrant, indicating their influence on PC1. Pulse, and segregate toward the top left quadrant with a small vector near the 0, perhaps suggesting that at this age, it was not an important variable to distinguish the impact of diet on kidney function.

The variables pointing in the same direction are positively correlated and the closer an observation is to a variable arrow, the higher the value of that variable for that observation. For example, Mean BUN Conc and Mean Urea Conc are positively correlated.

The PC1 explains most of the variance. Variables like Mean BUN Conc, Mean Urea Conc, Systolic, Diastolic, and MeanBP are significant contributors. Whereas PC2 explains the second-highest variance. Variables like Plasma Creatinine contribute significantly to PC2. Notably, while there is some overlap, there is also a visible separation indicating differences between the NPD and LPD groups.

Using the PCA biplot we found that the metabolic variables could distinguish samples between the F1 generations of parents fed an NPD or LPD. The variables Mean BUN Conc and Mean Urea Conc are strong contributors to the principal components and, hence, important for explaining the variance in the data. The overlapping confidence ellipses, however, suggest that while there are differences, the groups are not entirely distinct, at least not at this age. This was expected since we analyzed animals before or at the onset of hypertension.

Both LDA and PCA analyses support the presence of distinct physiological profiles between the NPD and LPD groups, driven primarily by differences in metabolic markers, although some overlap indicates that the groups are not entirely distinct. These findings reinforce the influence of parental dietary protein intake on the renal and cardiovascular health of the offspring.

## Discussion

Our study demonstrates the significant impact of a parental low-protein diet on kidney development and function in offspring, highlighting the long-term consequences of prenatal nutrition on renal function. Our findings report a reduced developmental capability, and morphological defects in the kidneys of offspring from the parental LPD group. Histological examination indicated an abnormal nephrogenic zone, dilated tubules, low glomerular counts, and the presence of fluid-filled cysts in P0 and 12-week-old F1 offspring’s kidneys from the F0 parental LPD group (Figs. 2 and 3). These results align with previous studies, including our own, with mice [26], and others utilizing rats [15, 16, 25, 38] and show reduced nephron endowment and structural abnormalities in response to prenatal LPD.

The prenatal environment is vital in determining proper fetal development and the long-term health of the offspring, affecting their risk of developing kidney and cardiovascular diseases in the future [9, 10, 19, 24]. Parental malnutrition is a major cause of intrauterine growth restriction, which in turn hampers fetal development. A maternal deficient diet during gestation leads to poor development of the placenta and the fetus. Consequently, it results in low birth weight and kidney weight. Both are risk factors for hypertension, and kidney disease later in life [19–23].

Our observations of low birth weight, reduced kidney size and morphological defects underscore the critical role of adequate protein intake during pregnancy for proper nephrogenesis and kidney health. These observations align with the ideas of Barker and Brenner on the fetal origin of adult disease [5, 6, 19, 21, 39, 40] Previous research effectively linked maternal poor nutrition with IUGR and low birth weight as a risk factor for the development of noncommunicable diseases later in life [7–9, 11]. Maternal and paternal protein deficiency can cause adult diseases, including cardiovascular and renal diseases [30, 41–45]. Our study underscores the importance of adequate nutrition during gestation and the need for careful monitoring of children born with low birth weight to prevent hypertension and chronic kidney disease later in life. We demonstrated that significant kidney damage accumulates even before the onset of hypertension (Figs. 2 and 3). Our data is supported by the observation of the Dutch and Chinese famine cohorts in which adults whose parents experienced famine during gestation developed hypertension and chronic kidney disease at higher rates than expected [46–51].

Previously, we applied single-cell RNA sequencing to study the effects of parental LPD on embryonic kidney development. We found that exposure to an LPD during fetal development led to a reduction of approximately 25% in nephron numbers at birth. Mechanistically, parental LPD led to molecular changes in the transcription of genes associated with cell proliferation, differentiation, and metabolism. These changes disrupted the developmental trajectory of kidney progenitor cells, ultimately culminating in oligonephropathy at birth [26]. In this study, we expanded our analysis to investigate the effects of LPD on postnatal kidney function and morphology at P0 and 12 weeks of age.

The offspring of parents fed an LPD tend to develop hypertension later in life [15, 27, 33, 45, 52]. However, hypertension itself is a marker of and can promote kidney damage. Therefore, our study focused on earlier time points, before a significant clinical rise in blood pressure, aiming to identify the primary impact of diet on kidney morphology, and not secondary damage due to hypertension.

We chose to analyze 12-week-old animals because this age marks the onset of increased blood pressure, which characterizes hypertension. The analysis of hemodynamic parameters showed that at this age there were no significant differences between the NPD and LPD groups in terms of pulse rate, systolic blood pressure, diastolic blood pressure, and mean blood pressure. Previous studies using rats (> 16 weeks) have reported hypertension in offspring exposed to prenatal LPD [25, 34, 52–54]. This hypertensive response appears multifaceted, with regards to the kidneys, it appears to be partially mediated by fetal exposure to excess maternal glucocorticoids [53, 55], oligonephropathy [37, 40, 56], and defects in the protein machinery responsible for nephron formation [26, 34, 57].

In our dataset, only two male mice (2/12) show increased blood pressure. This was an interesting observation due to reasons; first, it is generally accepted that men are at greater risk for cardiovascular and renal disease than are age-matched, premenopausal women, as reviewed previously [58]. Second, it shows that 12 weeks was indeed the threshold time to observe the deterioration of kidney function in the offspring from parental LPD that could be distinguished from NPD offspring by LDA analysis, but not enough deterioration that led to an increase in mean systolic blood pressure above 140 mmHg.

The metabolic panel analysis of 12-week-old mice born from F0 exposed to LPD during gestation indicates trends towards elevated levels of kidney function markers such as urine albumin, plasma creatinine, and mean urea concentration in the LPD group. Elevated urine albumin and plasma creatinine levels suggest compromised glomerular filtration and potential early signs of kidney dysfunction. These findings are consistent with the hypothesis that a prenatal deficient diet impairs renal development, predisposing offspring to hypertension and chronic kidney disease [19, 20, 40].

We performed a correlation analysis between metabolic and hemodynamic markers and found distinct patterns in the relationships between NPD and LPD groups. Notably, the LPD group showed stronger correlations among metabolic markers and between these metabolic markers and hemodynamics variables. Thus, indicating that small alterations in the metabolic panels reflected decreased kidney function which paved the way for increased blood pressure later. The alterations in both kidney metabolic panel and hemodynamic have been studied before after the establishment of hypertension [25, 37, 53, 54, 59]. Our work examined the mice before the instauration of hypertension and found an alteration in the metabolic kidney panel, confirming that this panel is more sensitive to detect the onset of kidney disease. This data can help to fine-tune treatment interventions before the instauration of hypertension, thus helping to preserve kidney function.

The LDA and PCA further helped to understand the differences between the NPD and LPD groups based on physiological measures. The LDA showed a clear separation between the groups along the first linear discriminant axis (LD1), indicating distinct physiological profiles influenced by prenatal diet. The PCA biplot revealed significant contributions of variables such as mean BUN concentration and mean urea concentration to the variance in the data, underscoring their importance in elucidating the importance of parental diet in offspring kidney function. These multivariate analyses highlight the utility of advanced statistical techniques in elucidating the complex interactions between diet and health outcomes.

Overall, our study underscores the profound impact of a parental low-protein diet on kidney development and function in offspring, with significant morphological, metabolic, and regulatory implications. These findings contribute to the growing body of evidence linking prenatal nutrition to long-term health outcomes and emphasize the need for adequate maternal nutrition to ensure optimal renal and cardiovascular health in the next generation. Future studies should focus on elucidating the molecular mechanisms underlying these effects and exploring potential therapeutic interventions to mitigate the adverse outcomes associated with prenatal LPD. Lastly, our study emphasizes that even subclinical alteration can be informative about kidney function and assist with earlier intervention to preserve kidney health.

## Electronic supplementary material

Below is the link to the electronic supplementary material.

Supplementary Material 1: Supp. Figure 1: Effect of Parental Low-Protein on Kidney Morphology in Newborn Pups (P0) H&E histology of P0 kidneys from offspring of parents fed an NPD. A normal cortical structure such as nephrogenic zone with several nascent nephrons (red arrows) and adjacent tubular structures, glomeruli (black G) with preserved Bowman space can be observed, (A, B and C). Kidney histology of P0 pup from LPD parents. The kidney section shows altered morphology, including abnormal nephrogenic zone, fluid-filled cysts (yellow C), and abnormal kidney structure such as dilated tubules, Glomeruli (black G), (D, E and F). Notice that the nephrogenic zone is less crowded with nuclei (blue), and more spaced with the extracellular matrix, suggesting an expansion of the stromal cells. Scale bar: 100 µm

## Data Availability

No datasets were generated or analysed during the current study.
