# Supplementary material for: Early metabolic and hemodynamic indicators of kidney dysfunction in mice offspring from parental low protein diet
Source: Mol Cell Pediatr. 2024 Oct 16;11:11. doi: 10.1186/s40348-024-00184-8 (PMC11480283; doi:10.1186/s40348-024-00184-8)

**Supp. Figure 1: Effect of Parental Low-Protein on Kidney Morphology in Newborn Pups (P0)** H&E histology of P0 kidneys from offspring of parents fed an NPD. A normal cortical structure such as nephrogenic zone with several nascent nephrons (red arrows) and adjacent tubular structures, glomeruli (black G) with preserved Bowman space can be observed, (A, B and C). Kidney histology of P0 pup from LPD parents. The kidney section shows altered morphology, including abnormal nephrogenic zone, fluid-filled cysts (yellow C), and abnormal kidney structure such as dilated tubules, Glomeruli (black G), (D, E and F). Notice that the nephrogenic zone is less crowded with nuclei (blue), and more spaced with the extracellular matrix, suggesting an expansion of the stromal cells. Scale bar: 100 µm


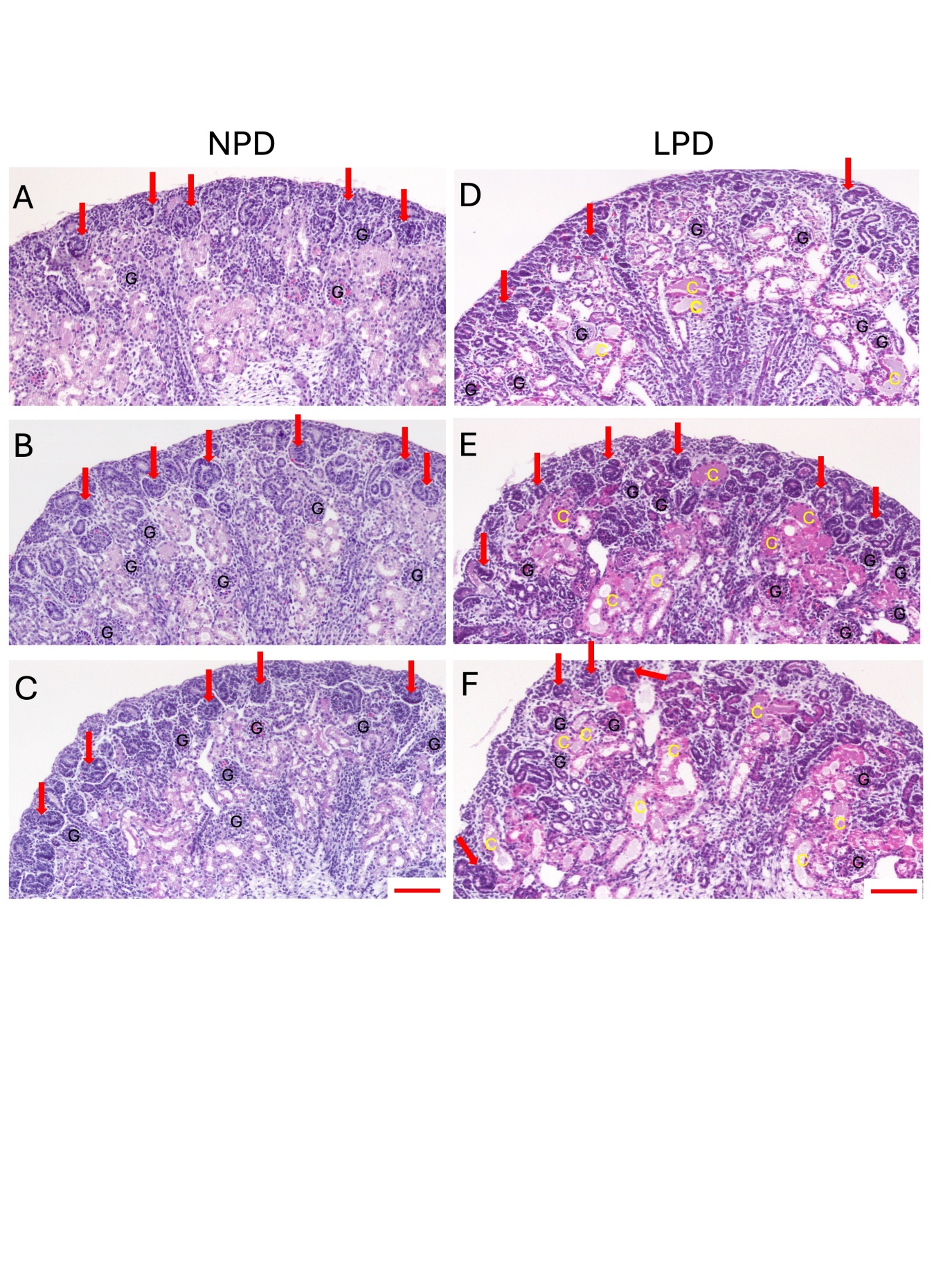

Supplement: Supplementary file 1 — Supplementary Material 1: Supp. Figure 1: Effect of Parental Low-Protein on Kidney Morphology in Newborn Pups (P0) H&E histology of P0 kidneys from offspring of parents fed an NPD. A normal cortical structure such as nephrogenic zone with several nascent nephrons (red arrows) and adjacent tubular structures, glomeruli (black G) with preserved Bowman space can be observed, (A, B and C). Kidney histology of P0 pup from LPD parents. The kidney section shows altered morphology, including abnormal nephrogenic zone, fluid-filled cysts (yellow C), and abnormal kidney structure such as dilated tubules, Glomeruli (black G), (D, E and F). Notice that the nephrogenic zone is less crowded with nuclei (blue), and more spaced with the extracellular matrix, suggesting an expansion of the stromal cells. Scale bar: 100 µm [file 40348_2024_184_MOESM1_ESM.docx]
